# Orientation toward humans predicts cognitive performance in orang-utans

**DOI:** 10.1038/srep40052

**Published:** 2017-01-09

**Authors:** Laura A. Damerius, Sofia I. F. Forss, Zaida K. Kosonen, Erik P. Willems, Judith M. Burkart, Josep Call, Birute M. F. Galdikas, Katja Liebal, Daniel B. M. Haun, Carel P. van Schaik

**Affiliations:** 1 Anthropological Institute and Museum, University of Zurich, Switzerland; 2 School of Psychology and Neuroscience, University of St. Andrews, United Kingdom; 3 Department of Archaeology, Simon Fraser University, Burnaby, BC, Canada; 4 Department of Education and Psychology, Freie University Berlin, Germany; 5 Leipzig Research Center for Early Child Development, University of Leipzig, Germany

## Abstract

Non-human animals sometimes show marked intraspecific variation in their cognitive abilities that may reflect variation in external inputs and experience during the developmental period. We examined variation in exploration and cognitive performance on a problem-solving task in a large sample of captive orang-utans (*Pongo abelii* & *P. pygmaeus*, N = 103) that had experienced different rearing and housing conditions during ontogeny, including human exposure. In addition to measuring exploration and cognitive performance, we also conducted a set of assays of the subjects’ psychological orientation, including reactions towards an unfamiliar human, summarized in the *human orientation index* (HOI), and towards novel food and objects. Using generalized linear mixed models we found that the HOI, rather than rearing background, best predicted both exploration and problem-solving success. Our results suggest a cascade of processes: human orientation was accompanied by a change in motivation towards problem-solving, expressed in reduced neophobia and increased exploration variety, which led to greater experience, and thus eventually to higher performance in the task. We propose that different experiences with humans caused individuals to vary in curiosity and understanding of the physical problem-solving task. We discuss the implications of these findings for comparative studies of cognitive ability.

Human intellectual performance is known to be strongly affected by developmental inputs^1^,^2^,^3^. However, similar effects in nonhuman primates have received far less attention. So far, the possible effect of experience on cognitive abilities in non-human primates has mainly been studied by examining the two extreme cases of deprivation and enculturation. Physical and social deprivation have been reported to cause strong negative outcomes on cognitive development in primates; especially maternal separation has been shown to result in both short- and long-term socio-cognitive consequences^4^,^5^,^6^,^7^,^8^,^9^,^10^,^11^. The opposite extreme in rearing environment is enculturation. Especially among great apes, enriched socio-cultural inputs, in the form of extensive interactions with humans, result in enhanced physical cognitive skills^12^,^13^,^14^ (see also ref. 15), but particularly in improved socio-cognitive and communicative abilities^16^,^17^,^18^,^19^,^20^. Both deprivation and enculturation therefore demonstrate that extreme social influences shape the cognitive abilities of nonhuman primates.

Even without being deprived or enculturated, captive primates also show intraspecific variability in cognitive abilities^21^,^22^, but the sources of this variability remain largely unstudied and it remains unclear whether the same social processes are involved. In particular, we don’t know to what extent variable rearing histories affect cognitive performance across individuals under non-extreme conditions, and if so whether they do so through their effect on psychological variables such as motivation to solve problems, responses to novelty and exploration style.

The aim of the present study was, first, to examine in a sample of more than 100 captive orang-utans (*Pongo abelii* and *P. pygmaeus*) how variation in captive management regimes and individual rearing histories affects psychological variables, such as human orientation, motivation and exploration style, and second, to examine whether these factors explain variation in cognitive performance in a tool-use task. Our sample contained orang-utans from a wide range of rearing backgrounds: both zoo groups, including mother- and hand-reared individuals, and individuals housed in rehabilitation stations who were wild-born but subsequently experienced captivity for variable periods of time, some as pets, before entering the rehabilitation station. Importantly, the individuals were all currently kept in captivity, allowing similar individual testing conditions. This provided us with an excellent opportunity to test the strength of the long-term effects of early rearing conditions on exploration styles and cognitive performance.

Because our sample included individuals from both zoos and rehabilitation stations, we could also test the independent effect of current housing conditions. Zoos and rehabilitation stations differ in their purpose of holding captive apes. Zoo apes have lived all their lives in a similar and stable environment, mostly together with their mothers and in intense contact with both knowledgeable conspecifics and human caretakers, with additional exposure to human strangers on a daily basis. Virtually all zoos also offer additional enrichment in the form of various foraging activities. In rehabilitation stations the purpose is very different. Some individuals may have experienced traumatic events in their past (capture and loss of mother, sometimes accompanied by injury) and thus need to recover physically and mentally. The stations’ ultimate aim is to return individuals to the wild. They consequently avoid bringing about a too close attachment to humans or exposing them to artificial enrichment devices. Moreover, most rehabilitating apes lack the close bond to their mother and are instead housed in peer groups.

Reactions to an unfamiliar human have previously been used to measure temperament in great apes^23^ and physiological distress responses in monkeys^24^,^25^. In order to estimate the underlying psychological consequences of human-related experiences that might explain variation in exploration and cognitive performance, we developed a new measure, the human orientation index (henceforth: HOI; see Methods for details). This measure was developed because the effect of captivity on cognitive abilities^26^,^27^ seems to reflect a major psychological change. First, because in multiple species, reduced neophobia has been shown to influence problem-solving skills^28^,^29^,^30^,^31^, and because captive orang-utans show strongly increased interest in novel items relative to their wild counterparts^32^ (as do other species^27^,^33^), we assessed the response to novelty across individuals with different captive experiences. Second, because the effect of captivity on cognitive performance is particularly pronounced in the context of tool use^34^,^35^,^36^,^37^,^38^,^39^, we assessed cognitive performance using a novel tool-use task including multi-step problem-solving with high ecological validity: the honey-tool task. In order to prevent variation in human orientation from confounding cognitive performance during the testing, no humans were present during the tests, which were video-recorded.

## Results

### Cognitive performance

**Figure 1:**
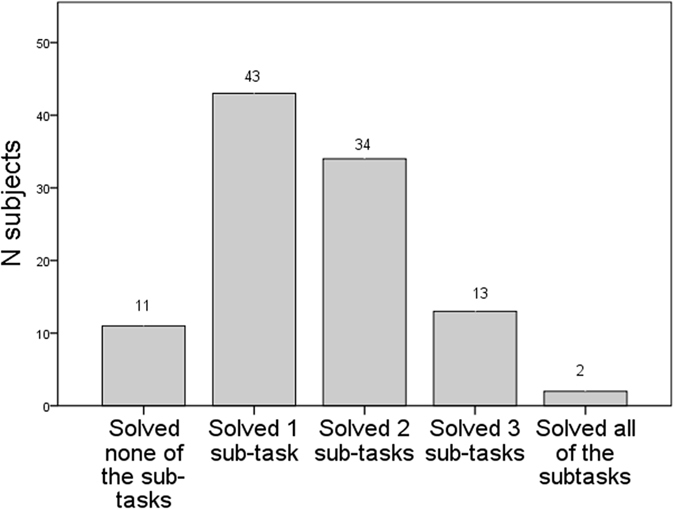
Honey tool-task performance. Frequency of subjects that solved zero to all subtasks.

**Figure 2:**
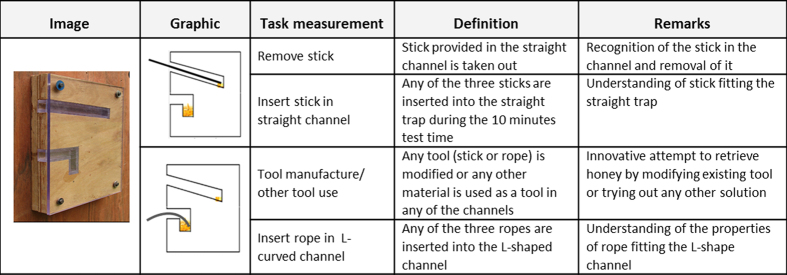
Overview of the design, structure and measurements of cognitive performance. The honey tool-task offered two problems to solve: an upper channel with a stick solution and lower channel with a rope solution.

For the honey tool-task, we found large variation in cognitive performance among the 103 orang-utans (Fig. 1). Eleven individuals did not solve a single sub-task. The modal and median score on total performance was 1 out of a maximum of 4. The four different cognitive measurements of the honey tool-task varied significantly in difficulty. In order to examine whether the ability to solve the different sub-tasks was cumulative, we applied a Guttman scale, which showed a reproducibility coefficient of close to one (0.97). This strongly suggests that the ability to solve the more difficult sub-tasks was nested within the performance of the other sub-tasks: 90% of the 103 individuals performing the honey tool-task fitted the applied Guttman scale of the four different sub-tasks (see Methods and Fig. 2 for details). As expected, ‘remove stick’ was the easiest, ‘insert the stick in the straight channel’ was next, followed by ‘making a tool’, whereas ‘inserting the rope in the curved channel’ was most difficult (Fig. 2, Sub-task) for the orang-utans in this study. Due to this variation in difficulty, we treated the four levels of cognitive performance as an ordinal variable in further analyses.

We next tested whether background and housing conditions, species, sex, and age could explain the variation in cognitive performance on the honey tool-task. Table 1 shows the results of a binomial Generalized Linear Mixed Model (GLMM) of the honey tool-task performance, with the response variable being whether or not a subject solved each sub-task. The GLMM allowed us to control for repeated observations in each facility and on each individual. The results revealed that the human orientation index (HOI) was a good predictor of the ability to solve the task (B ± SD = 0.227 ± 0.08, Z = 2.699, P = 0.007; Table 1), as was the latency to touch unfamiliar food (B ± SD = −0.034 ± 0.01, Z = −2.263, P = 0.024; Table 1) and a novel object (B ± SD = −0.025 ± 0.01, Z = −2.123, P = 0.034; Table 1). The less neophobic the individuals, the more likely they were to solve the task. When we removed the novelty responses from the analysis, the HOI remained the main predictor of performance (Table S1, supplementary material). Because an individual’s age and the time it had spent in captivity were strongly correlated, only the factor age was considered for the analysis, but it did not influence task performance. Subtle differences between enclosures, which might affect the ease of access to the apparatus, were controlled for in the analysis, but again did not contribute to explaining the variation in cognitive performance. Perhaps surprisingly, none of the other possible variables (sex, species [Sumatran or Bornean], and the various background and current housing conditions of the orang-utans) predicted performance; they also had no influence on task performance when HOI was excluded from the analyses (Table S2, Supplementary material).

### Exploration behaviour underlying cognitive performance

**Figure 3:**
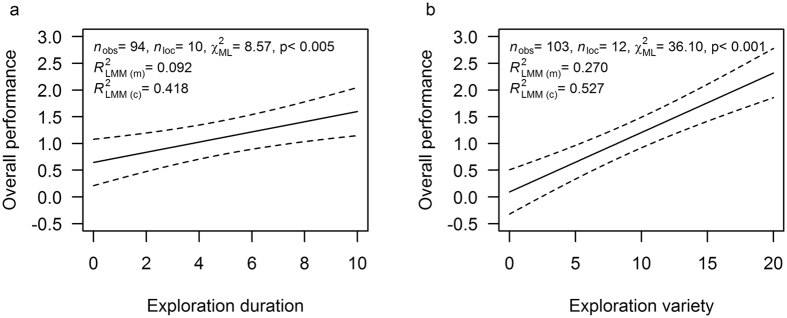
The relationships between exploration behaviour and the overall cognitive performance. (**a**) Overall performance in relation to the exploration duration. Individuals that explored longer were significantly better problem solvers (N_obs_ = 94, N_location_ = 10, χ^2^_ML_ = 08.57, P = 0.005). (**b**) The overall task performance in relation to the total variety of exploration actions (N_obs_ = 103, N_location_ = 12, χ^2^_ML_ = 36.10, P < 0.001).

**Figure 4:**
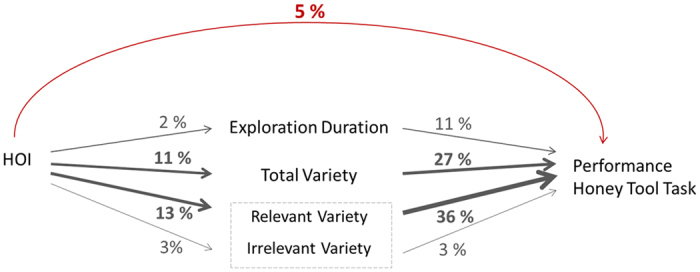
Graphical illustration of the relationship between the human orientation Index (HOI), the cognitive performance and exploration duration and variety, in context to each other. The total variety can be divided into relevant and irrelevant variety. The figure also indicates the percentage of variation estimated by the pseudo *R*^2^ for linear mixed effects margins that is explained by each factor. The thickness of the arrows accentuates the strength of the influence.

The orang-utans’ performance on the tool-use task was best accounted for by exploration variety, which explained 27% of the variation (Figs 3b and 4), whereas exploration duration predicted 9% of the variation (N_obs_ = 94, N_location_ = 10, χ^2^_ML_ = 08.57, *R*^2^_LMM(m)_ = 0.092, P = 0.005, Figs 3a and 4). However, the HOI only explained 5% of cognitive performance (χ^2^_ML_ = 6.21, P < 0.05).

### The effect of human orientation on exploration

**Figure 5:**
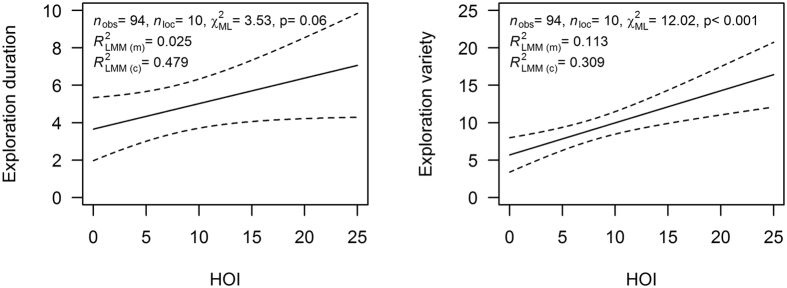
The relationships between the human orientation index (HOI) and exploration behaviour showed significant dependencies in Linear-Mixed-Effect-Models (LMM) that controlled for repeated observations in each facility. (**a**) Exploration duration in relation to HOI-index (N_obs_ = 94, N_location_ = 10, χ^2^_ML_ = 3.53, *R*^2^_LMM(m)_ = 0.025, P = 0.06). (**b**) Total variety of exploration actions in relation to the HOI (N_obs_ = 94, N_location_ = 10, χ^2^_ML_ = 12.02, *R*^2^_LMM(m)_ = 0.1113, P < 0.001).

A Linear Mixed-Effects Model (LMM) analysis of the subjects’ exploration of the apparatus, which controlled for repeated measurements in each facility, showed that HOI accounted for 11% of the variation of the total exploration variety (N_obs_ = 94, N_location_ = 10, χ^2^_ML_ = 12.02, *R*^2^_LMM(m)_ = 0.1113, P < 0.001, Figs 4 and 5b). In addition, there was a trend showing that individuals with a higher HOI explored the apparatus longer than those with a lower HOI (N_obs_ = 94, N_location_ = 10, χ^2^_ML_ = 3.53, *R*^2^_LMM(m)_ = 0.025, P = 0.06, Fig. 5a), although the HOI explained a mere 2% of the variation in exploration duration (Fig. 4). This low proportion is not surprising, because the most successful orang-utans, and thus the ones with high HOI values, stopped exploring once they found the solution to the problem and spent their remaining time extracting honey.

Random exploration of the apparatus per se does not necessarily raise the chances of finding the solution. Individuals who can distinguish between relevant and irrelevant parts of the apparatus should be more successful (Supplementary Table S3). We therefore also investigated the separate effects of exploration variety directed at the relevant or irrelevant areas of the apparatus. We calculated marginal pseudo-*R*^2^ values to estimate the proportion of variation explained by the fixed effects in our model. The HOI explained 13% of the variation of relevant exploration (χ^2^_ML_ = 13.67, P < 0.001, Fig. 4 and Supplementary Table S4), which subsequently accounted for 36% of the variation in cognitive performance. In contrast, HOI explained only 3% of the irrelevant exploration (χ^2^_ML_ = 2.93, P = 0.087, Fig. 4), which accounted for only 3% of performance in the task. The effect of a higher HOI was thus primarily on the amount of exploration and especially on the diversity of exploration on relevant parts of the task, with the latter explaining 36% of cognitive performance. Furthermore, neither housing- nor background/ rearing history had any effect on the exploration of the apparatus (Supplementary Table S4).

Additionally, using a Linear Mixed-Effect Model (LMM), controlling for each subject’s housing location, we compared the exploration style of the most successful individuals, the 10 subjects who solved the most difficult problem of ‘inserting the rope in the curved channel’ (‘ropers’), to the other non-successful individuals (‘non-ropers’). There was no difference in exploration duration between ropers and non-ropers. However, the ropers differed significantly from non-ropers in their exploration variety (P = 0.012). Ropers not only showed a greater diversity of explorative actions, but also a far greater diversity of exploration on relevant parts of the apparatus (p < 0.001; note that solving the rope solution is itself not counted as relevant exploration). Moreover, the ropers’ HOI was 29.7% (and significantly) higher than that of non-ropers (χ^2^_ML_ = 4.06, P < 0.05).

### Evaluating the human orientation index

**Figure 6:**
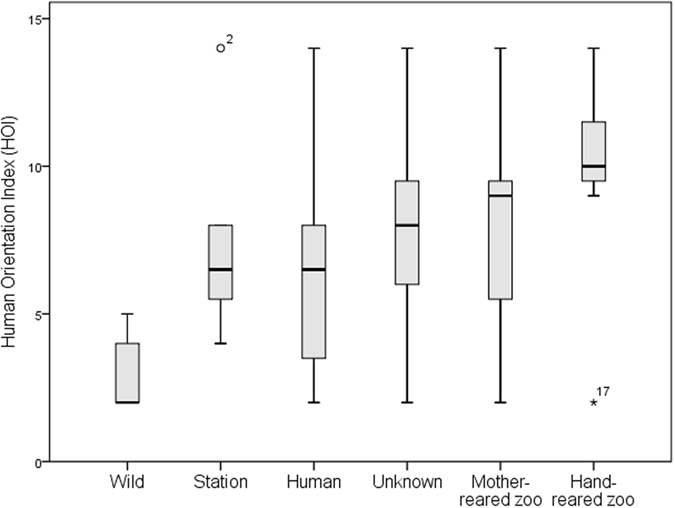
The human orientation index (HOI) in relation to background history. Groups of individuals with different background histories differed slightly in their human orientation.

Given the large effect of the HOI on exploration style, we examined whether the different background categories determined an individual’s human orientation index. ‘Wild’ individuals strongly diverged from any other category in that they took longer or did not respond at all to the novel food and novel object (Supplementary Fig. 1) and by showing significantly lower HOI values (Kruskal-Wallis test: N = 95, P = 0.002, Fig. 6). However, pairwise comparisons of each background category (controlling for age, sex and species as well as repeated observations from each study location and correction of P-values for multiple comparisons using Tukey) revealed no significant differences in HOI between the background categories (Supplementary Table: S5).

The reaction toward novel humans might also be the result of several other factors, such as a response to any novelty or to social beings (human or orang-utan). Therefore, we also examined the links between HOI and the three novelty response experiments (concerning novel food, novel objects and novel conspecifics). Results of a Linear Mixed-Effects Model (LMM) evaluated the relation between the HOI and two other novelty response tasks, the novel-object and the novel-food task. The HOI was not explained by the latency to touch either the novel food (B ± SD = −0.057 ± 1.12, *df* = 78.83, t = 6.401, P = 0.332; Table 2) or the novel toy (B ± SD = 0.008 ± 0.02, *df* = 81.9, t = 0.457, P = 0.649; Table 2), whereas the latter two were correlated (Spearman’s rho: r = 0.314, N = 98, P = 0.002, 2-tailed). Neither did the exploration duration of the novel toy explain the variation in HOI (B ± SD = 0.006 ± 0.01, *df* = 81.99, t = 0.584, P = 0.561; Table 2). Thus, the HOI did not simply reflect a positive response to novelty per se. To test whether the HOI represents a general interest in social beings, and thereby a higher social motivation in general or whether it describes the interest in humans specifically, we performed an additional social-interest-task with a subset of individuals of one rehabilitation station (N = 28, see Method section). The HOI did not seem to measure a general social interest, since the duration spent in close proximity to a novel human did not correlate with the time spent in close proximity to novel conspecifics (Spearman’s rho: r = 0.198, N = 28, P = 0.312, two-tailed).

## Discussion

This study represents one of the largest systematic individual-level comparisons of cognitive tool-using abilities in apes, involving 13 different captive groups in both zoos and rehabilitation stations in which none of the individuals were deprived or enculturated. As expected, the latency to touch novel food or objects, and thus reduced neophobia, was an important independent predictor of task performance (Table 1), as has been found in other studies^28^,^29^,^30^,^31^. However, we also found that variation in problem-solving skills in the honey tool-task was equally predicted by persistent and varied explorative behaviour, which in turn was highly influenced by the orang-utans’ psychological orientation as assessed with the human orientation index, HOI (Table 1). We suggest the following biologically most plausible causal cascade, backed up by a series of analyses: Human orientation mainly influenced both the orang-utans’ motivation to explore and the nature of their exploration, and consequently affected their understanding of the problem-solving task, and thus their success in solving it.

The reaction towards humans could have several dimensions, other than the mere interest in humans, and our results allowed us to characterize the nature of the Human orientation Index (HOI) in more detail. A high HOI does not simply reflect the expectation of food that is provided regularly by humans, because HOI varied extensively and all these orang-utans depended on humans for their food. The different background categories also showed higher variability in their HOI than in their novelty response (Fig. 6 and Supplementary Fig. S1). Moreover, if it were mere food expectation, variation in HOI should be associated with caretakers that provide the daily food supply rather than random strangers. Finally, the HOI does not reflect general novelty response or general social interest, as it was not correlated with the approach latency to novel food nor objects (Table 2), nor with interest in novel conspecifics. Therefore the effect was human-specific and increased the motivation to explore, expressed as increased duration and variety of exploration (Fig. 5a and b).

The HOI thus captures a fundamental psychological change that is induced by human contact. The different background categories overlapped largely in their HOI (Supplementary Table S5), implying that each individual’s specific nature and experience of human contact is more influential than the human exposure time per se. However, our sample included a few wild individuals who had spent their whole immature period in natural habitat and showed very low human orientation compared to most other conspecifics housed in zoos and rehabilitation stations, independent of the time they had spent at the station (Fig. 6). This indicates that the change caused by humans can only happen at an early age, suggesting a sensitive period for social inputs. Since this kind of psychological orientation is absent in nature^32^, we can ask which natural process is being mimicked or modified by human contact. The answer is remarkably simple: humans replace the role of the mother and other conspecific experts, and the rich variety of artefacts provided by humans enriches their physical environment. In their natural niche, orang-utans as well as other primates are prone to attend to their mother and other expert conspecifics and learn necessary skills socially^40^,^41^,^42^,^43^,^44^,^45^. Exploration plays a crucial role in skill acquisition in the wild, but virtually all exploration is socially facilitated, allowing orang-utans to overcome intrinsic neophobia^32^.

Given the identification of human orientation, rather than rearing conditions, as the key determinant of cognitive ability in captive apes, it makes sense to revisit the role of deprivation and enculturation. Because deprivation involves the complete loss of any role models, whereas enculturation involves the presence of far richer social inputs by more actively engaged role models than under normal conditions, one could argue that the degree of human orientation may largely explain the whole spectrum of cognitive performance among great apes. This perspective also explains why enculturated apes outperform others not just in socio-cognitive skills, but also in physical cognitive skills^19^,^46^.

The social triggering of the engagement with artefacts is highly influential in human child development^47^,^48^.

Studies within the field of comparative psychology have documented non-human primates’ tendency to attend to humans^17^,^49^ and acknowledged the improvement in learning cognitive tasks due to human contact in captive settings^50^,^51^. Systematic species comparisons of primates’ attention structure toward humans are rare. Nonetheless, in 1916, Yerkes^52^ already suggested that the qualitatively better cognitive performance of an orang-utan compared to monkeys was due to the ape’s social attention to human actions. Our results thus support previous suggestions^39^,^53^,^54^ that early exposure to humans and human artefacts presents a broader range of opportunities for exploration resulting in increased innovativeness in captive apes. Over time, the accumulating experience resulting from attention to humans leads to improved problem-solving ability, provided the exposure to humans is early in life. In conclusion, human orientation at least partly explains the phenomenon that captive primates that are exposed to both conspecific and human role models experience increased opportunities for socially induced exploration and learning (cf. ref. 55).

Our detailed analyses revealed that the HOI influenced an individual’s duration and especially its variety of exploration (Fig. 5a and b), which subsequently explained cognitive performance (Fig. 3a and b, Fig. 4). Previous studies on hyenas^29^ and birds^56^ have also reported that the diversity of exploration actions influences innovativeness and problem-solving skills. However, in our study, individuals with a strong human orientation were more successful in the task, not only through their exploration diversity, but also by focusing on the relevant parts of the apparatus (Fig. 4), implying that they were better at recognizing the actual challenge presented in the honey tool-task. Importantly, these parts were relevant not because they directly led to the solution, but rather because exploration of these parts improved the animals’ understanding of the physical properties of the problem. For example, individuals that traced the honey channel from the outside of the glass obviously understood that there is honey inside, but were at that time not searching at the correct part of the apparatus, the channel entrance. Similarly, individuals poking with their finger into the curved channel may have gathered information on its length. Exploration can therefore be viewed as latent learning: it allows an animal to gather knowledge of the texture, the material, and the problem itself. Over time, then, individuals with a high HOI will gain more experience, which contributes to their focus on relevant aspects of the problem and hence problem-solving success.

The effects of the HOI on problem-solving success may have been so strong that they masked the effects of other factors. Thus, we found no differences between the two orang-utan species (*P. abelii* and *P. pygmaeus*), even though these were found when orang-utans with very similar backgrounds (all mother-reared zoo individuals) were compared on a range of cognitive tasks^57^.

This study documented strong effects of human orientation on problem-solving abilities, through its effects on response to novelty, motivation to explore, exploration persistence and ultimately experience. This finding suggests that it is just as impossible to design culture-free cognitive tests for primates as it is for humans. In this sense, tests of primate cognition are inevitably deeply anthropomorphic. However, once we have controlled for the subjects’ human-related histories and given that problem-solving ability is about dealing with unknown, novel problems the variation captured in these tests nonetheless reflects variation in intrinsic cognitive abilities and should be comparable within and across species. Therefore, we suggest the HOI may be a useful tool in standardizing comparisons across primates, especially studies concerning ape subjects with various background and human-related experiences. In future work, we will further disentangle the exact nature and causes of the HOI and address additional problem-solving domains.

## Methods

### Subjects and study facilities

Our total sample size involved 103 orang-utans: 68 *Pongo pygmaeus spp* and 35 *Pongo abelii* (Supplementary Table S6). Data collection on the zoo-housed sample took place at nine different European zoos between November 2012 and January 2015; all zoo data was collected by SF (Table 3). In total the zoo sample consisted of 41 individuals, of whom 31 were mother-reared and 10 whose own mother had either died or rejected the infant and were therefore hand-reared. They were cared for by human caretakers, within the zoo or partly within a human household, and subsequently returned to a group of zoo-living conspecifics (Table 4).

Data on 62 rehabilitation orang-utans were collected between June 2012 and June 2014 by LD and ZK, supported by a trained assistant, Andreas Wendl. In Borneo data collection took place at two rehabilitation stations, both situated in Central Kalimantan (Table 3). In Sumatra data collection took place at two sites of the same station: the quarantine station and at the release site (Table 3).

Depending on available background information the sample collected at the rehabilitation stations was further divided into the following groups: Wild, Station, Human and Unknown. Individuals were assigned to these four groups depending on the estimated age at arrival at a rehabilitation station (based on tooth eruption patterns) and their previous history with humans (Table 4). Table 4 shows that infants are usually caught when very young because then they are still easy to handle and thus most attractive as pets.

### Housing conditions

In the zoos, individuals were housed in mixed-aged groups of conspecifics ranging from four to 12 individuals in standard indoor enclosures during the day, and mostly separated individually or in pairs into sleeping quarters for the night. Most zoos also provide the orang-utans with a larger outdoor enclosure. At each zoo, animal keepers are in daily close contact with the orang-utans, providing them with food but also with diverse enrichment activities. Zoo visitors were additionally in daily visual contact with the orang-utans.

In the rehabilitation stations the housing situations were more heterogeneous (see Table 3). They differed according to the standards and capabilities of each facility and the age, sex and background of the individuals. All orang-utans in this study were held in solitary enclosures at the time of the study, except for one station housing 28 individuals socially in groups of 2–6 individuals. In general contact with humans was reduced to caretakers cleaning and feeding several times a day and veterinary care as needed. Each enclosure had simple enrichment devices, such as ropes, and several times a week the subjects received extra food-related enrichment or leafy branches. In some facilities, small infants lived in a nursery with other orang-utan infants and human caretakers serving as replacement mothers. These infants had daily extended contact with their foster-mothers and other human caretakers. Most individuals had access to forest vegetation at some stage during their time at the station.

### Human Orientation Index

To capture any psychological variation caused by time in captivity and human-related experiences, we assessed the degree to which each individual reacted toward a novel human. The Human Orientation Index (HOI) contained the following components: reactions and proximity to a human stranger during two conditions. Each subject was tested individually, except for a few cases where the mother was tested with its dependent offspring to avoid inducing stress for both. In the zoos the test took place either in the home enclosure or in the sleeping quarters if individuals were more easily separated there. In the rehabilitation stations, individuals were either transported to single compartments for testing or were directly tested in their home enclosure. The test was performed by a local man, unknown to the orang-utans and dressed in black.

The total test lasted for one minute and was composed of two consecutive conditions, each lasting 30 seconds. In the first condition the man approached and positioned himself approximately one meter in front of the enclosure where the subject was located and remained standing with his body oriented laterally (perpendicularly) to the subject. In the second condition the man turned around to face the orang-utan and tried to establish eye contact. The whole test was video recorded and during the entire test no other human was present.

Reactions and proximity to the man for the first two seconds of first sight were coded from the videos. For each condition we scored the proximity to the man in the following way: 0 = the orang-utan positioned itself as far away as possible; 1 = the orang-utan was more than one meter away from the human; 2 = the orang-utan was within one meter from the human; and 3 = the orang-utan placed itself as close to the human as possible.

We also scored the very first behavioural reaction of the orang-utan for each condition as follows: 0 = a negative reaction, defined as: retreat, stress vocalization, pilo-erection, nervous swinging or turning away from the human; 1 = a neutral reaction, defined as resting, moving calmly or play behaviour; 2 = a positive reaction, if the orang-utan approached the human; and 3 = an actively positive reaction, if the orang-utan begged (either by using lips or hands), tried in any other active way to contact the human or attempted to trade objects from the enclosure for food.

Furthermore, since the measurements listed above were based upon the first reaction of each condition only, we also scored whether any active contact behaviour occurred during the 30 seconds of each condition. This was to ensure catching the possible substantial interest in humans, when the surprise had waned.

Thus, in total HOI consisted of all the summed behavioural reactions combined with the proximity to a human stranger, with the eventual score ranging from zero to 14. In our sample, scores ranged from 2 to 14. Furthermore, we also measured the time in seconds a subject spent within one meter of the human stranger throughout the whole test and found that this independent time measurement of proximity was strongly correlated with the HOI-index (Spearman’s correlation, two-tailed: r_s_ = 0.600, N = 96, P < 0.001). Given that an individual can be in close proximity and not move throughout the time of the test, but nevertheless not show any active response behaviour, we used the summarized index of both behavioural reactions and proximity scores, which also generated more resolution to the various responses within our sample, than simply proximity latency data would.

The logistics in one of the rehabilitation stations allowed us to use a sub-sample of 28 individuals to test for social interest in unfamiliar conspecifics. In this sub-sample we measured the time of close proximity to two other unknown orang-utans of equal sex, when these were present in a neighbouring enclosure to the subject, which allowed us to disentangle social orientation per se to that from interest in humans.

### Response to novelty

We performed two separate tests to assess individual variation in novelty response. First, we examined how each subject reacted to novel food. In the zoos, the novel food was blue mash potato served with black olives on top. Zoo orang-utans receive a broad diet with many types of human food, but blue items are not common and olives were new to all individuals. In the rehabilitation stations we used purple rice or purple mash potato with dried purple sweet potato pieces. Second, we introduced a novel toy in the form of a wooden board equipped with six differently coloured, rotatable tennis balls. For both tasks, we recorded the latency from task begin (when set up was completed and individual was in max. 1 m distance to the apparatus) until first touch, as well as exploration duration of the novel toy. Maximum test duration was two minutes for both tasks, and each subject was tested individually.

### Experimental cognitive task – The honey tool-task

In order to evaluate cognitive performance we used a naturalistic task, which required no pre-training trials and could therefore easily be applied to all individuals. The honey tool-task allowed us to assess physical cognition of tool-use at multiple levels from very basic understanding of the apparatus and tools to high innovativeness. The task involved a wooden box measuring 50 cm × 80 cm × 5 cm, whose front was covered by a transparent Plexiglas^®^ or Macrolon^®^ plate (Fig. 2). The upper part of the box contained a straight channel (30 cm × 5 cm) where a wooden stick (40 cm) with its tip dipped in honey had been inserted. Below the straight channel, the box had a L-shaped channel (15 cm × 10 cm) with its bottom part filled with honey. The honey was visible to the subjects through the glass, but both channels were too long to reach the honey with their fingers. Moreover, the wooden stick could not be used to reach the honey in the L-shaped channel. In addition, below the test apparatus we provided each subject with two more wooden sticks and three pieces of rope. The ropes were too short to reach the end of the straight channel but long enough to retrieve the honey at the bottom of the L-shaped channel.

We measured multiple aspects of the orang-utans’ responses to the apparatus, which was presented to them in the absence of any humans. To estimate cognitive ability, we measured the following actions: 1) removing the pre-inserted stick from the straight channel; 2) inserting any of the three available sticks into the straight channel during total test time; 3) tool manufacturing, defined as an attempt to modify the provided tools and/ or the use of any other item found by the subjects as a tool for the honey channels; 4) inserting the rope tool into the L-shaped channel. We coded each action separately as yes or no, depending on whether or not a subject performed it (Fig. 2).

We also recorded detailed data on any exploration actions during the problem-solving task. These were divided into two main categories: relevant and irrelevant. Relevant exploration concerned the channels, and thus the actual problem to be solved. Any other explorative acts directed toward the test apparatus itself, the board or table was coded as irrelevant exploration. For both categories of exploration, we measured the frequency, the duration, and the variety (see Supplementary Table S3 for definitions).

### Experimental procedure

In all tasks (novelty response tests, the HOI test and the honey tool-task), only those individuals participated who could easily be separated without showing any signs of separation-induced stress. Accordingly, sample size across the different tests varied from 94 to 103. In the honey tool-task each orang-utan was tested individually, except for two mothers who were tested with their dependent offspring, in which case the offspring did not participate in the task. All subjects were naïve to this test apparatus and we performed no training trials. All subjects were tested only once. Zoo individuals were tested in their smaller sleeping enclosures where they could be separated from the group. Testing in the rehabilitation stations took place in the home enclosures, since most individuals were housed alone. For the 28 socially housed individuals, additional testing enclosures were available. The individuals were brought to the testing enclosures separately and only stayed there for the time of testing. We therefore incorporated the identity of the rehabilitation station as one factor in our analyses. The problem-solving task lasted a maximum of 10 minutes. Because the individuals’ experience with humans was so variable, we conducted the problem-solving task without the presence of an experimenter to avoid possible effects on the subject’s participation and attention during cognitive testing (cf. ref. 58). The task was video recorded with one to two SONY HDR-CX200 handy cameras, depending on angle of the cameras. None of the orang-utans were food-deprived for the task. In the rehabilitation stations, the honey tool-task was presented to the subjects on a large board right outside of their enclosure, and subjects could easily reach out toward the problem-solving task. In all but one zoo, the apparatus was also presented outside the enclosure. However due to the logistics and narrower mesh size in the zoos, we presented the apparatus closer to the mesh with a slight angle but less accessible to the orang-utans compared to the rehabilitation station setting. In one zoo, we presented the honey box within the test enclosure, with orang-utans having full access to the apparatus. We therefore incorporated accessibility of the test apparatus as one factor in our analyses.

### Data extraction and statistical analyses

All videos were imported into Mangold interact 9.7, in which all detailed behaviours of both cognitive performance measurements as well as exploration acts were coded by SF and LD. We used IBM SPSS Statistics 20 to perform inter-observer reliability tests on every behavioural measure that occurred during the honey-tool task. For the zoo sample, 20% of the videos were coded by both observers and yielded a Cohen’s Kappa of 0.842 (N_events_ = 1020, P < 0.001), which is considered very good. From the rehabilitation sample, 16% of the videos were coded by both observers and yielded a good inter-rater agreement (Cohen’s Kappa: 0.721, N_events_ = 1020, P < 0.001). Also the behavioural responses and the proximity measurements that generated the Human Orientation Index was coded in Mangold interact 9.7 by SF and a trained research assistant AS and reached a good inter-observer reliability value of 0.853 (Cohen’s Kappa: N_responses_ = 52, P < 0.001). LD and SF reached an inter-observer reliability value (IOR) of 0.782 (Cohen’s Kappa: N_responses_ = 66, P < 0.001) in a sample of over 26.6% of the zoo-videos. LD and and AS reached a substantial agreement within the sample of rehabilitation orang-utans of 0.701 (Cohen’s Kappa: N_responses_ = 185, P < 0.001).

Further statistical analyses were performed in R version 3.2.3^59^,^60^ using the ‘lme4’^61^ and ‘MUMIn’^62^ packages. Individual scores on each of the four measures of cognitive performance (exhibit: Yes/No) were modeled by a binomial Generalized Linear Mixed Model (GLMM). We incorporated each individual’s HOI-score, age, sex, species, accessibility of apparatus, and ontogenetic background (rearing and housing condition), along with the measure of cognitive performance (Table 1) as fixed factors, and controlled for repeated observations on each individual within its respective facility by specifying this as a nested random effect. For categorical predictor variables with more than two levels, we manually specified planned contrasts. For accessibility of the test apparatus and the measure of cognitive performance (both ordinal predictor variables), we conducted polynomial trend analyses, while for ontogenetic background we set orthogonal contrasts to compare: 1) wild subjects against all other subjects, 2) subjects from rehabilitation centres against zoo subjects, 3) within rehabilitation centres, subjects from unknown provenance against all other subjects, 4) within rehabilitation centres, human-reared subjects against centre-reared subjects, and finally 5) within zoos, hand-reared subjects against mother-reared subjects. To examine each individual’s relationship between the HOI scores, novelty response, exploration variables and performance, we used Linear-Mixed-Effect-Models (Figs 2, 3 and 4).

### Ethical statement

All experiments fully complied with the ethical guidelines of each study facility (zoological garden/ rehabilitation station) and were respectively approved by the research manager and/or head of each facility. We confirm that according to the Swiss Animal Welfare legislation our animal experiments are considered with the severity grade 0 (no harm). The experimental protocols for the rehabilitation stations were approved by the Animal Welfare office of the University of Zurich, the Scientific Advisory Board of the BOS Foundation (Bornean Orangutan Survival), the research managers and head of the stations of Sumatran Orangutan Conservation Programme (SOCP) and Orangutan Foundation International (OFI), and the Indonesian Ministry of Research and Technology (RISTEK). Moreover, all zoo experiments were supported by research committee of the British and Irish association for zoos and aquariums, BIAZA.

## Additional Information

**How to cite this article**: Damerius, L. A. *et al*. Orientation toward humans predicts cognitive performance in orang-utans. *Sci. Rep.* **7**, 40052; doi: 10.1038/srep40052 (2017).

**Publisher's note:** Springer Nature remains neutral with regard to jurisdictional claims in published maps and institutional affiliations.

## Supplementary Material

Supplementary Material

## Figures and Tables

**Table 1:** Generalized Linear Mixed Model of overall performance in the honey tool-task.

	*B*	*SE*	*Z*	*p value*
(Intercept)	−2.588	1.33	−1.943	0.052
**Human Orientation Index**	0.227	0.08	2.699	0.007**
**Novel food: time until touch**	−0.034	0.01	−2.263	0.024*
**Novel object: time until touch**	−0.025	0.01	−2.123	0.034*
**Novel object: exploration duration**	0.003	0.01	0.433	**0.665**
Age	−0.006	0.03	−0.232	0.817
SEX (male)	0.124	0.51	0.243	0.808
Species (Sumatra)	−0.094	0.59	−0.16	0.873
Background				
Wild vs. Rest	−0.173	0.27	−0.639	0.523
Rehab vs. Zoo	−0.402	0.29	−1.395	0.163
Unknown vs. Rehab.Rest	0.073	0.20	0.367	0.714
Human vs. Station (within rehabilitation station)	−0.479	0.48	−1.008	0.314
Mother vs. Hand (within zoo)	0.365	0.51	0.722	0.470
Accessibility (trend analysis)				
Linear	0.786	0.76	1.04	0.298
Quadratic	0.228	0.89	0.257	0.797
Cubic	−0.189	0.89	−0.213	0.831
Sub-task (trend analysis)				
** Linear**	−4.969	0.74	−6.737	<0.001***
** Quadratic**	1.554	0.44	3.559	<0.001***
Cubic	−0.205	0.33	−0.626	0.531

Note: The model is controlling for repeated observations on each facility and individual. The performance in the honey tool-task was binary measured. The Analysis included 88 individuals in 9 different zoos/rehab stations, totalling 352 observations, *χ*^*2*^ = 226.27, *P* < 0.001, P-values below 0.05 appear in bold. Parameter estimates from a binomial GLMM, predicting the probability of an animal solving the task.

**Table 2:** Linear Mixed-Effects model of the Human Orientation Index controlling for repeated observations on each facility.

	*Estimate*	*SE*	*df*	*t value*	*p value*
(Intercept)	7.155	1.12	58.15	6.401	<0.001***
Novel food					
Latency touch	−0.057	0.06	78.83	−0.977	0.332
Novel Object					
Latency touch	0.001	0.02	81.9	0.457	0.649
Exploration duration	0.006	0.01	81.99	0.584	0.561

*Note: N observations* = *85, N place* = *9, X*^*2*^_*ML*_ = *3.556, P* = *0.314,* P-values below 0.05 appear in bold.

**Table 3:** Overview of subjects and facilities.

Study Facility	N	Species	Age Range	Social Housing	Test Location	Time Period
Zoo 1	6	*P. pygmaeus*	6–43	Mixed-age group	Sleeping quarter	Apr-May 2013
Zoo 2	8	*P. pygmaeus*	13–52	Mixed-age group	Sleeping quarter	Jan-Feb 2014
Zoo 3	4	*P. abelii*	5–13	Mixed-age group	Smaller enclosure	Nov 2013
Zoo 4	4	*P. pygmaeus*	13–31	Mixed-age group	Sleeping quarter	Jan 2015
Zoo 5	4	*P. abelii*	8–23	Mixed-age group	Sleeping quarter	Nov-Dec 2012
Zoo 6	5	*P. abelii*	9–49	Mixed-age group	Sleeping quarter	Mar 2013
Zoo 7	6	*P. abelii*	5–25	Mixed-age group	Test enclosure	Mar 2014
Zoo 8	2	*P. pygmaeus*	18–20	Mixed-age group	Sleeping quarter	Feb 2015
Zoo 9	2	*P. pygmaeus*	14–36	Mixed-age group	Sleeping quarter	Jan-Feb 2013
Rehab. Station 1	5	*P. abelii*	3–6	Solitary	Home enclosure	Apr-Jun 2013
Rehab. station 2	18	*P. pygmaeus*	6–17	Solitary	Home enclosure	May-Jun 2014
Rehab. station 3	28	*P. pygmaeus*	8–14.5	Peer group	Test enclosure	June-Sep 2012
Rehab. station 4	11	*P. abelii*	5–25	Solitary	Home enclosure	Oct 2012–Mar 2013

**Table 4:** Categories of subjects and their background histories.

Background during early development	N	Age Range (years)	Current Housing	Years in Captivity	Human Exposure	Remark
Wild	5	10–25	Rehabilitation station	0–7	Minimal	Majority of life in natural habitat. Arrived at a rehabilitation center as adolescents or adults, eventually to be translocated to a new natural habitat.
Station	8	5–11	Rehabilitation station	4–10	Mainly human raised.	Minimum 80% of life in rehabilitation station. Arrived at station as dependent offspring at the age of 1.5 year or younger.
Human	16	3.5–14	Rehabilitation station	0–9	Minimum 6 months with humans	Arrived at rehabilitation station older than 1.5 years of age. Background history with human contact (minimum 6 months), e.g. pet
Unknown rehab	33	3–17	Rehabilitation station	0.5–14	Unknown before arrival at station	No background information. Arrived at station between 2 and 7 years of age and spent possibly large part of the developmental phase in captivity.
Mother-reared Zoo	31	5–52	Zoo	Whole life	All life within human care	Nursed by own mother within a zoo. All life in captivity with exposure to human caretakers and visitors.
Hand-reared Zoo	10	13–43	Zoo	Whole life	Human hand nursing and all life within human care	Nursed by human caretakers either at the zoo or in human households. All life in captivity with exposure to human caretakers and visitors.
