# Supplementary material for: Orientation toward humans predicts cognitive performance in orang-utans
Source: Sci Rep. 2017 Jan 9;7:40052. doi: 10.1038/srep40052 (PMC5220318; doi:10.1038/srep40052)
Supplement: Supplementary Material [file srep40052-s1.pdf]

Supplementary material

Orientation toward humans predicts cognitive performance in orang-utans

[1]Laura A. Damerius* & [1]Sofia I. F. Forss* (* = shared first authorship), [1]Zaida K. Kosonen, [1]Erik P. Willems, [1]Judith M. Burkart, [2]Josep Call, [3]Birute M. Galdikas, [4]Katja Liebal, [5]Daniel B. Haun and [1]Carel P. van Schaik

[1]*Anthropological Institute and Museum, University of Zurich, Switzerland*

[2]*School of Psychology and Neuroscience, University of St. Andrews, United Kingdom*

[3]*Department of Archaeology, Simon Fraser University, Burnaby, BC, Canada*

[4]*Department of Education and Psychology, Freie University Berlin, Germany*

[5]*Leipzig Research Center for Early Child Development, University of Leipzig, Germany*

Supplementary Table S1: Parameter estimates from a binomial GLMM, predicting the probability of an animal solving the task when excluding independent variables for the novelty response tests.

Generalized Linear Mixed Model of overall performance in the honey tool-task.

Parameter estimates from a binomial GLMM, predicting the probability of an animal solving the task.

	B	***SE***	***Z***	***p value***
**Human Orientation Index**	0.243	0.09	2.679	**0.007 ****
Age	-0.018	0.03	-0.582	0.561
SEX (male)	-0.619	0.53	-1.158	0.247
Species (Sumatra)	0.295	0.60	0.484	0.628
<u>Background</u>				
Wild vs. Rest	-0.212	0.24	-0.890	0.373
Rehab vs. Zoo	-0.131	0.30	-0.436	0.663
Unknown vs. Rehab.Rest	0.160	0.22	0.730	0.466
Human vs. Station (within rehabilitation station)	-0.489	0.52	-0.933	0.351
Mother vs. Hand (within zoo)	0.383	0.52	0.573	0.465
<u>Accessibility (trend analysis)</u>				
Linear	1.211	0.87	1.395	0.163
Quadratic	1.209	0.97	1.249	0.212
Cubic	0.099	0.97	0.103	0.918
<u>Sub-task (trend analysis)</u>				
**Linear**	-5.046	0.72	-6.981	**<0.001*****
**Quadratic**	1.512	0.42	3.567	**<0.001*****
Cubic	-0.149	0.33	-0.457	0.648

Note: Analysis included 94 individuals in 10 different zoos/rehab stations, totalling 376 observations, χ^2_{ML}= 220.60, P< 0.001

Supplementary Table S2: Parameter estimates from a binomial GLMM, predicting the probability of an animal solving the task. Analysis without the HOI.

General Linear Mixed Model of overall performance in the honey tool-task.

Parameter estimates from a binomial GLMM, predicting the probability of an animal solving the task.

	B	***SE***	***Z***	***p value***
(Intercept)	-0.609	0.91	-0.670	0.503
Age	0.001	0.03	0.023	0.981
SEX (male)	-0.638	0.51	-1.252	0.210
Species (Sumatra)	0.186	0.56	0.332	0.740
<u>Background</u>				
Wild vs. Rest	-0.360	0.22	-1.604	0.109
Rehab vs. Zoo	-0.115	0.26	-0.447	0.655
Unknown vs. Rehab.Rest	0.221	0.21	1.041	0.298
Human vs. Station (within rehabilitation station)	-0.613	0.51	-1.201	0.230
Mother vs. Hand (within zoo)	0.157	0.41	0.381	0.704
<u>Accessibility (trend analysis)</u>				
Linear	0.910	0.81	1.118	0.263
Quadratic	1.499	0.83	1.803	0.071 .
Cubic	0.120	0.82	0.146	0.884
<u>Sub-task (trend analysis)</u>				
Linear	-4.905	0.66	-7.478	**<0.001** ***
Quadratic	1.509	0.39	3.830	**<0.001** ***
Cubic	0.036	0.31	0.118	0.906

Note: The model is controlling for repeated observations on each facility and individual. The performance in the honey tool-task was binary measured. The Analysis included 103 individuals in 12 different zoos/rehab stations, totalling 412 observations, χ^2= 236.43, P< 0.001

Supplementary Table S3: Detailed list of all different exploration actions coded as relevant and irrelevant exploration during the time an individual engaged with the honey tool-task.

Relevant Exploration Actions	Irrelevant Exploration Actions
Insert stick into L-shaped channel	Hit (with any body parts) test apparatus or board
Insert finger into L-shaped channel	Hit (with any body parts) table on which apparatus is presented
Insert finger into I-shaped channel	Pull/ Push test apparatus, plexiglas, screws
Insert rope into I-shaped channel	Pull/ Push table or board on which apparatus is presented
Trace/ Poke finger outside glass of either I- or L-shaped channel	Touch test apparatus, plexiglas, screws (elsewhere but channels)
Trace/ Poke stick outside glass of either I- or L-shaped channel	Touch table or board on which apparatus is presented
Tool modification/ manufacture	Poke stick at apparatus, plexiglas, screws (elsewhere but channels)
Insert any other tool than stick or rope into L-shaped channel	Poke stick at board/table on which apparatus is presented
Insert any other tool than stick or rope into I-shaped channel	Touch sticks on the floor or besides test apparatus
Poke or push stick at opening of L-shaped channel	Touch ropes on the floor or besides test apparatus
Poke or push stick at opening of I-shaped channel	
Poke or push finger at opening of L-shaped channel	
Poke or push finger at opening of I-shaped channel	

Supplementary Table S4: Linear Mixed-Effects Model of relevant exploration variety controlling for repeated observations on each facility.

	B	*SE*	*df*	*t value*	*p value*
(Intercept)	1.209	1.41	48.66	0.857	0.396
**HOI**	0.221	0.05	80.07	4.049	**<0.001 *****
Age	0.014	0.03	80.84	0.414	0.680
SEX (male)	-0.059	0.55	79.52	-0.106	0.916
Species (Sumatra)	0.946	0.87	6.31	1.084	0.318
<u>Background</u>					
Wild vs. Rest	0.162	0.25	75.37	0.652	0.517
Rehab vs. Zoo	0.356	0.33	42.06	1.063	0.294
Unknown vs. Rehab.Rest	0.408	0.23	80.76	1.746	0.085 .
Human vs. Station (within rehabilitation station)	-0.257	0.53	79.81	-0.483	0.630
Mother vs. Hand (within zoo)	0.314	0.58	65.78	0.544	0.589
<u>Accessibility (trend analysis)</u>					
Linear	0.316	1.11	12.64	0.285	0.780
Quadratic	0.467	1.13	23.52	0.411	0.685
Cubic	1.540	1.04	77.21	1.486	0.141

Note: The model is controlling for repeated observations on each facility. The analysis was totalling 94 observations in 10 different zoos/rehab stations, $\chi^2 = 30.91$, $p < .0005$

Supplementary Table S5: Pairwise comparisons of the Human Orientation Index (HOI) between the different background and rearing categories.

Pairwise comparisons of HOI between different background categories, controlling for age, sex, island and repeated observations from each zoo and rehabilitation station.				
	B	***SE***	***Z***	***p value***
Hand-reared zoo - Station	1.649	2.03	0.814	0.960
Human - Station	-1.452	1.35	-1.078	0.877
Mother-reared zoo - Station	-0.605	1.62	-0.372	0.999
Unknown - Station	-0.279	1.28	-0.217	1.000
Wild - Station	-3.568	2.46	-1.450	0.670
Human – Hand-reared zoo	-3.102	1.82	-1.707	0.497
Mother-reared zoo – Hand-reared zoo	-2.254	1.50	-1.499	0.637
Unknown – Hand-reared zoo	-1.928	1.74	-1.106	0.864
Wild – Hand-reared zoo	-5.217	2.70	-1.931	0.354
Mother-reared zoo - Human	0.847	1.35	0.626	0.987
Unknown - Human	1.174	0.94	1.249	0.792
Wild - Human	-2.116	2.25	-0.941	0.927
Unknown – Mother-reared zoo	0.326	1.25	0.260	1.000
Wild – Mother-reared zoo	-2.963	2.41	-1.227	0.805
Wild - Unknown	-3.289	2.41	-1.468	0.658
P-values corrected for multiple comparisons using Tukey.				

Supplementary Table S6 study subjects.

Individual	Age in years at testing	years in captivity	species	current housing	Category
**Amin**	6	0.5	*Pongo abelii*	Reha-Station	unknown background
**Amos**	13	13.0	*Pongo pygmaeus*	Zoo	Mother reared
**Andalas**	3	0.5	*Pongo abelii*	Reha-Station	unknown background
**Anette**	30	30.0	*Pongo abelii*	Zoo	Mother reared
**Ari**	10	8.0	*Pongo pygmaeus*	Reha-Station	Background with humans
**Ayu**	6	0.5	*Pongo abelii*	Reha-Station	unknown background
**Bahruni**	10	0.5	*Pongo abelii*	Reha-Station	wild/ translocated
**Bambang**	10.5	5.0	*Pongo pygmaeus*	Reha-Station	Background with humans
**Batu**	14	14.0	*Pongo pygmaeus*	Zoo	Mother reared
**Bella**	14.5	10.0	*Pongo pygmaeus*	Reha-Station	unknown background
**Binti**	13	13.0	*Pongo pygmaeus*	Zoo	Human hand reared
**Budi**	8	8.0	*Pongo abelii*	Zoo	Mother reared
**Cane**	10	0.0	*Pongo abelii*	Reha-Station	wild/ translocated
**Cantik**	10	8.0	*Pongo pygmaeus*	Reha-Station	unknown background
**Ceky Chan**	6	0.0	*Pongo abelii*	Reha-Station	Background with humans
**Cherie**	18	18.0	*Pongo pygmaeus*	Zoo	Mother reared
**Cici**	15	10.0	*Pongo pygmaeus*	Reha-Station	unknown background
**Dagu**	28	28.0	*Pongo abelii*	Zoo	Mother reared
**Dana**	25	25.0	*Pongo abelii*	Zoo	Mother reared
**Dandim**	12	7.0	*Pongo pygmaeus*	Reha-Station	unknown background
**Dewa**	12	10.0	*Pongo pygmaeus*	Reha-Station	unknown background
**Djamuna**	13	13.0	*Pongo abelii*	Zoo	Mother reared
**Dokana**	25	25.0	*Pongo abelii*	Zoo	Mother reared
**Dora**	3.5	2.0	*Pongo abelii*	Reha-Station	Background with humans
**Duanne**	10	8.0	*Pongo pygmaeus*	Reha-Station	unknown background
**Edwin**	11.5	7.0	*Pongo pygmaeus*	Reha-Station	unknown background
**Embrie**	10	8.0	*Pongo pygmaeus*	Reha-Station	unknown background
**Franky**	7	0.5	*Pongo abelii*	Reha-Station	unknown background
**Friend**	5.5	3.5	*Pongo abelii*	Reha-Station	unknown background
**Gagak**	9.5	8.0	*Pongo pygmaeus*	Reha-Station	Reha-Station reared

**Galih**	10	7.0	*Pongo pygmaeus*	Reha-Station	Background with humans
**Gambira**	18	18.0	*Pongo pygmaeus*	Zoo	Mother reared
**Gina**	49	45.0	*Pongo abelii*	Zoo	Mother reared
**Gober**	25	5.0	*Pongo abelii*	Reha-Station	wild/ translocated
**Harry**	8.5	7.0	*Pongo pygmaeus*	Reha-Station	Reha-Station reared
**Hulu**	14	8.0	*Pongo pygmaeus*	Reha-Station	Background with humans
**Imas**	9.5	8.0	*Pongo pygmaeus*	Reha-Station	Background with humans
**Inou**	17	15.0	*Pongo pygmaeus*	Reha-Station	unknown background
**Ito**	6	6.0	*Pongo pygmaeus*	Zoo	Mother reared
**Jack2**	9	6.0	*Pongo pygmaeus*	Reha-Station	Background with humans
**Jacky**	5	1.0	*Pongo abelii*	Reha-Station	Background with humans
**Janu**	6	4.0	*Pongo pygmaeus*	Reha-Station	unknown background
**Jarot**	5	4.0	*Pongo abelii*	Reha-Station	Reha-Station reared
**Jarwo**	15	10.0	*Pongo pygmaeus*	Reha-Station	unknown background
**Jaya**	9	9.0	*Pongo abelii*	Zoo	Mother reared
**Jill**	8	7.0	*Pongo pygmaeus*	Reha-Station	Reha-Station reared
**Jose**	21	21.0	*Pongo pygmaeus*	Zoo	Mother reared
**Julius**	5	2.0	*Pongo abelii*	Reha-Station	Background with humans
**Karan**	10	8.0	*Pongo pygmaeus*	Reha-Station	unknown background
**Karen**	15	9.0	*Pongo pygmaeus*	Reha-Station	unknown background
**Kasmin**	10.5	9.0	*Pongo pygmaeus*	Reha-Station	Background with humans
**Kevin**	32	32.0	*Pongo pygmaeus*	Zoo	Human hand reared
**Kibriah**	36	36.0	*Pongo pygmaeus*	Zoo	Human hand reared
**Kila**	13	13.0	*Pongo abelii*	Zoo	Mother reared
**King**	17	10.0	*Pongo pygmaeus*	Reha-Station	unknown background
**Kraba**	12	10.0	*Pongo pygmaeus*	Reha-Station	unknown background
**Lanang**	11	10.0	*Pongo pygmaeus*	Reha-Station	Reha-Station reared
**Mali**	20	20.0	*Pongo pygmaeus*	Zoo	Mother reared
**Mandi**	14	14.0	*Pongo pygmaeus*	Zoo	Human hand reared
**Mawoto**	17	13.0	*Pongo pygmaeus*	Reha-Station	unknown background
**Maya**	5	5.0	*Pongo abelii*	Zoo	Mother reared
**MercedeS**	12	10.0	*Pongo pygmaeus*	Reha-Station	unknown background
**Natalia**	18	0.0	*Pongo abelii*	Reha-Station	wild/ translocated
**Natalie**	11	9.0	*Pongo pygmaeus*	Reha-Station	unknown background

**Niken**	16	14.0	*Pongo pygmaeus*	Reha-Station	unknown background
**Nonja**	36	36.0	*Pongo pygmaeus*	Zoo	Human hand reared
**Oracle**	8.5	7.0	*Pongo pygmaeus*	Reha-Station	Reha-Station reared
**Otong**	13	11.0	*Pongo pygmaeus*	Reha-Station	unknown background
**Padana**	16	16.0	*Pongo abelii*	Zoo	Mother reared
**Pilar**	11	9.0	*Pongo pygmaeus*	Reha-Station	Background with humans
**Pini**	25	25.0	*Pongo abelii*	Zoo	Mother reared
**Pongo**	15	15.0	*Pongo pygmaeus*	Zoo	Mother reared
**Radja**	52	51.0	*Pongo pygmaeus*	Zoo	Mother reared
**Raja**	13	13.0	*Pongo abelii*	Zoo	Mother reared
**Ramon**	18	18.0	*Pongo pygmaeus*	Zoo	Human hand reared
**Roma**	17	13.0	*Pongo pygmaeus*	Reha-Station	unknown background
**Rowland**	10	8.0	*Pongo pygmaeus*	Reha-Station	unknown background
**Runtu**	13	8.0	*Pongo pygmaeus*	Reha-Station	Background with humans
**Sabin**	9.5	9.0	*Pongo pygmaeus*	Reha-Station	Reha-Station reared
**Sandakan**	32	32.0	*Pongo pygmaeus*	Zoo	Mother reared
**Sari**	43	43.0	*Pongo pygmaeus*	Zoo	Human hand reared
**Sarimin**	11	9.0	*Pongo pygmaeus*	Reha-Station	unknown background
**Silvia**	49	48.0	*Pongo pygmaeus*	Zoo	Mother reared
**Sony**	16	12.0	*Pongo pygmaeus*	Reha-Station	unknown background
**Suaq**	5	5.0	*Pongo abelii*	Zoo	Mother reared
**Sule**	7	4.0	*Pongo pygmaeus*	Reha-Station	unknown background
**Summer**	13	13.0	*Pongo pygmaeus*	Zoo	Mother reared
**Suri**	5	3.0	*Pongo abelii*	Reha-Station	Background with humans
**Tanah**	5	5.0	*Pongo abelii*	Zoo	Mother reared
**Tao**	8	8.0	*Pongo abelii*	Zoo	Mother reared
**Temmy**	31	31.0	*Pongo pygmaeus*	Zoo	Human hand reared
**Toba**	18	18.0	*Pongo abelii*	Zoo	Mother reared
**Trio**	16	13.0	*Pongo pygmaeus*	Reha-Station	unknown background
**Ulin**	14	11.0	*Pongo pygmaeus*	Reha-Station	unknown background
**Vendel**	13	13.0	*Pongo abelii*	Zoo	Mother reared
**Victor**	10.5	9.0	*Pongo pygmaeus*	Reha-Station	Background with humans
**Victoria**	31	31.0	*Pongo pygmaeus*	Zoo	Human hand reared
**Walter**	23	23.0	*Pongo abelii*	Zoo	Mother reared

**Wattana**	18	18.0	*Pongo pygmaeus*	Zoo	Human hand reared
**Willy**	6	3.0	*Pongo abelii*	Reha-Station	Background with humans
**Winda**	12	7.0	*Pongo pygmaeus*	Reha-Station	wild/ translocated
**Yogi**	8	8.0	*Pongo pygmaeus*	Reha-Station	Reha-Station reared
**Zatarra**	10	8.0	*Pongo pygmaeus*	Reha-Station	unknown background

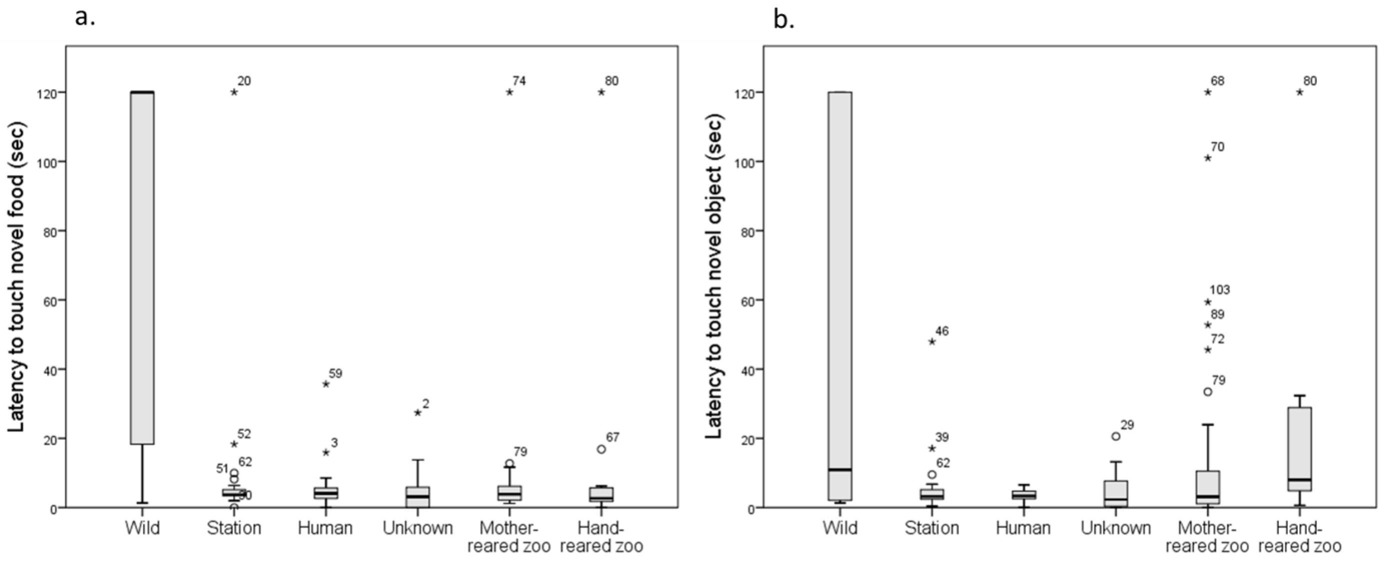

Figure S1: Novelty response across individuals of different background categories. (**a**) Latency to touch novel food and (**b**) Latency to touch a novel object.
